# The Drosophila Splicing Factor PSI Is Phosphorylated by Casein Kinase II and Tousled-Like Kinase

**DOI:** 10.1371/journal.pone.0056401

**Published:** 2013-02-20

**Authors:** J. Matthew Taliaferro, Dhruv Marwha, Julie L. Aspden, Daniela Mavrici, Nathalie E. Cheng, Lori A. Kohlstaedt, Donald C. Rio

**Affiliations:** 1 Department of Molecular and Cell Biology, University of California, Berkeley, California, United States of America; 2 Center for Integrative Genomics, University of California, Berkeley, California, United States of America; 3 California Institute for Quantitative Biosciences (QB3), University of California, Berkeley, California, United States of America; International Centre for Genetic Engineering and Biotechnology, Italy

## Abstract

Alternative splicing of pre-mRNA is a highly regulated process that allows cells to change their genetic informational output. These changes are mediated by protein factors that directly bind specific pre-mRNA sequences. Although much is known about how these splicing factors regulate pre-mRNA splicing events, comparatively little is known about the regulation of the splicing factors themselves. Here, we show that the *Drosophila* splicing factor P element Somatic Inhibitor (PSI) is phosphorylated at at least two different sites by at minimum two different kinases, casein kinase II (CK II) and tousled-like kinase (tlk). These phosphorylation events may be important for regulating protein-protein interactions involving PSI. Additionally, we show that PSI interacts with several proteins in *Drosophila* S2 tissue culture cells, the majority of which are splicing factors.

## Introduction

Alternative splicing of pre-mRNA transcripts is widespread among eukaryotes. It is currently believed that over 90% of human genes and over 60% of *Drosophila* multi-exon genes are alternatively spliced [1], [2]. The list of splicing events regulated by a particular protein in a particular cellular context is known for many factors, usually through the use of RNAi and splicing-sensitive microarrays or high throughput mRNA-seq [3], [4]. Using splicing-sensitive microarrays, PSI was found to regulate 43 splicing events in S2 cells [5].

PSI was originally identified as a protein factor necessary for the retention of the third intron of the P element transposon, leading to the production of a truncated protein [6], [7]. PSI, like its mammalian homologs contains four N-terminal KH-type RNA binding domains. This repression of splicing occurs through an interaction between a C-terminal region of PSI and the U1 snRNP 70 K subunit [8], [9] and also requires the presence of a PSI binding motif near the affected intron [10], [11]. PSI deletion mutants are embryonic lethal, and its protein-protein interaction with U1 snRNP 70 K is necessary for male fertility [12].

Although many studies have investigated the effects that splicing factors have on alternative splicing, few have looked at the regulation of the factors themselves. Many of these instances of regulation occur pre-translationally, often at the level of splicing. For example, many SR proteins regulate their own splicing as well as that of heterologous SR proteins in a way that shunts those transcripts into the NMD pathway [13], [14].

Some splicing factors are known to be post-translationally modified. These events can affect the RNA binding capabilities of the protein [15] as well as the assembly of higher-order structures like the spliceosome [16]. The spliceosomal proteins SAP155 and NIPP1 are phosphorylated, and this modification is necessary for their interaction [17]. SR proteins and other splicing factors are highly phosphorylated *in vivo* [18]. Additionally, the RS domain of the splicing factor ASF/SF2 greatly affects the protein and RNA binding capabilities of the protein, and is necessary for splicing [15], [19].

We have identified two phosphorylation sites on PSI by mass spectrometry and identified two kinases that phosphorylate the N-terminus of PSI. These phosphorylation events may play a role in the ability of PSI to interact with other proteins. Additionally, we have identified several interaction partners of PSI, suggesting that PSI is present in cells a member of large ribonucleoprotein complexes.

## Results

### PSI is Phosphorylated in vivo

**Figure 1:**
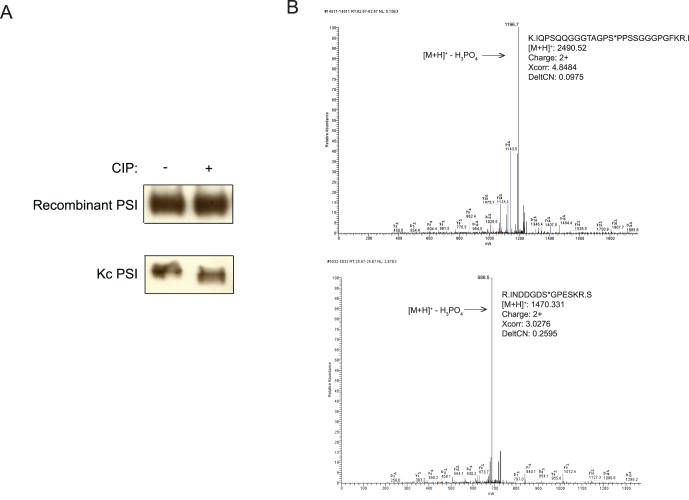
Biochemcial fractionation and analysis of the PSI kinase. A) Purified recombinant PSI and PSI purified from Kc cells was treated with calf intestinal phosphatase (CIP) and then visualized by immunoblotting. B) MS2 spectra identifying phosphopeptides found in PSI. B and Y series ions and neutral loss of phosphate are indicated. Inset: sequence of the phosphopeptide and SEQUEST statistics. MS3 spectra and corresponding spectra of unmodified peptides are given in supplemental Figure 1.

**Figure 4:**
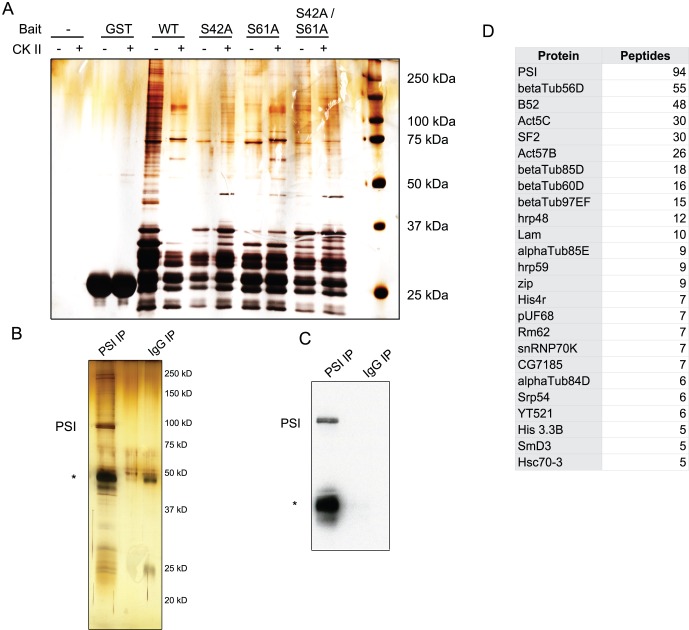
Protein-protein interactions of PSI. A) GST pulldown assay using PSI mutant proteins. GST-PSI fusion proteins carrying the serine to alanine PSI mutations were phosphorylated using purified human casein kinase II and incubated with Kc nuclear extract. The resulting glutathione resin eluates were analyzed by silver staining and mass spectrometry. B) Silver stain of PSI and interacting proteins following anti-polyoma and anti-PSI immunoprecipitations. The asterisk indicates antibody heavy chain. C) Immunoblot analysis of (B) using anti-PSI antibody. D) Mass spectrometry analysis of (B). Proteins identified as interacting with PSI and the number of peptides observed for each protein are listed.

Using a Polyoma (also known as Glu-Glu) tagged version of PSI, we purified PSI from *Drosophila* Kc cells. Interestingly, PSI purified from *Drosophila* cells migrated on SDS-PAGE gels as a doublet (Figure 1A, Figure 4B) while recombinant PSI purified from *E. coli* migrated as a single species (Figure 1A). We reasoned that PSI phosphorylation events occurring in *Drosophila* cells could be responsible for the doublet. Consistent with this idea, treatment of PSI purified from Kc cells with calf intestinal phosphatase (CIP) collapsed the doublet to a faster migrating band while having no effect on the migration of recombinantly produced PSI (Figure 1A).

To characterize this apparent phosphorylation, we digested purified endogenous Drosophila PSI with multiple proteases and analyzed the resulting peptides by multidimensional chromatography/mass spectrometry. The resulting data covered 84.5% of the sequence to an average depth of 10 observations per peptide. Manual evaluation of the spectra assigned to phosphopeptides confirmed two phosphorylation sites at Ser 42 and Ser 61(Figure 1B). Spectra showing phosphorylation at Ser42 were measured 51 times in four different peptides. Spectra showing phosphorylation at Ser61 were measured 5 times in two different peptides. Spectra of the corresponding unmodified peptides were measured 86 and 5 times, respectively. The characteristics of the unmodified spectra supported the interpretation of the modified spectra (Supplemental Figure 1).

The global phosphoproteomic analyses of Drosophila embryos by Zhai et al. [20] also identified Ser 42 and Ser 61 as phosphorylation sites. A third site, Ser 85, identified in that study was not detected on our analysis. Inspection of the supporting data from Zhai et al for Ser 85 showed that the CID spectrum contained no neutral loss peak; consequently, the identification is likely to be a false positive.

### PSI is Phosphorylated in Drosophila Cells by Casein Kinase II

**Figure 2:**
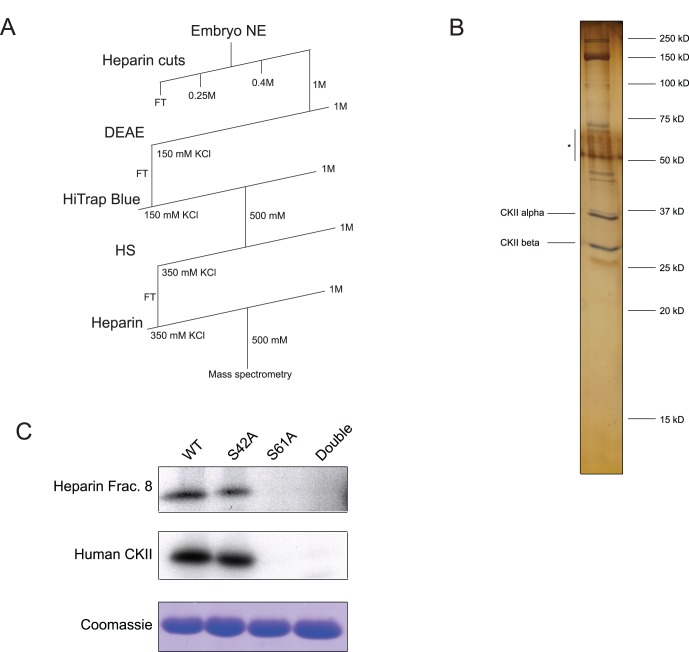
Biochemical purification of Drosophila casein kinase II. A) Purification strategy for endogenous casein kinase II. B) Protein composition of peak fraction of activity from the final heparin column visualized by SDS-PAGE and silver-staining. Species identified as casein kinase II alpha and beta are labeled. Bands labeled with an asterisk correspond to contaminating keratin. C) *In vitro* kinase assay of PSI mutant proteins. Serine to alanine PSI mutant proteins were phosphorylated *in vitro* using the peak fraction of activity from the final heparin column and using purified recombinant human casein kinase II (NEB P6010S). Assays were visualized using autoradiography, and, to ensure equal protein loading, coomassie staining.

In order to identify the protein kinase or kinases responsible for the observed phosphorylations of PSI, we used chromatographic fractionation of *Drosophila* embryo nuclear extract and followed PSI-phosphorylating activity using recombinant PSI and radioactive gamma-^32^P-ATP as substrates (Figure 2A). To simplify the purification and exclude the phosphorylation of other residues, we used an N-terminal fragment of PSI that contained only the first 95 amino acids, including the two residues, Ser 42 and Ser 61, that we identified as being phosphorylated by mass spectrometry (Figure 1B).

After five purification steps, we analyzed the protein content of the peak fraction of PSI kinase activity by SDS-PAGE and silver staining (Figure 2B). We detected two prominent protein species migrating at approximately 37 and 30 kDa that appeared to be at stoichiometric levels with each other. We excised these bands, as well as several other prominent bands, from the gel and performed mass spectrometry. The 37 and 30 kDa bands were identified as the alpha and beta subunits, respectively, of casein kinase II. Importantly, no other known protein kinases were identified in this fraction. We calculated the enrichment in specific activity contained in this fraction to be approximately 12,000-fold relative to the starting nuclear extract.

Casein kinase II is a tetramer composed of two catalytic 40 kDa alpha subunits and two regulatory 25 kDa beta subunits [21] and has many known protein targets in *Drosophila* [22], [23], [24], [25]. Analytical gel filtration chromatography of the peak activity fraction of kinase activity showed an approximate size of 135 kDa for the kinase, consistent with a tetramer composed of two 40 kDa and two 25 kDa subunits (data not shown).

Casein kinase II has a preferred recognition motif of *SXX(D/E) [26]. One of the identified phosphorylation sites, Ser 61, lies within this motif (*SGPE). We therefore hypothesized that casein kinase II was phosphorylating Ser 61. To confirm this, we made serine-to-alanine mutants at each phosphorylation site (Ser42 and Ser61), as well as a double mutant. We used both the peak activity fraction from the casein kinase II purification as well as purified recombinant human casein kinase II to phosphorylate these mutant PSI substrates in vitro (Figure 2C). The mutation of Ser 61 to alanine completely abolished to ability of casein kinase II to phosphorylate the substrate while the mutation of Ser 42 to alanine had little, if any, effect. Taken together, these data indicate that Ser 61 in PSI is a casein kinase II phosphorylation site.

### PSI is Phosphorylated in Drosophila Cells by Tousled-like Kinase

**Figure 3:**
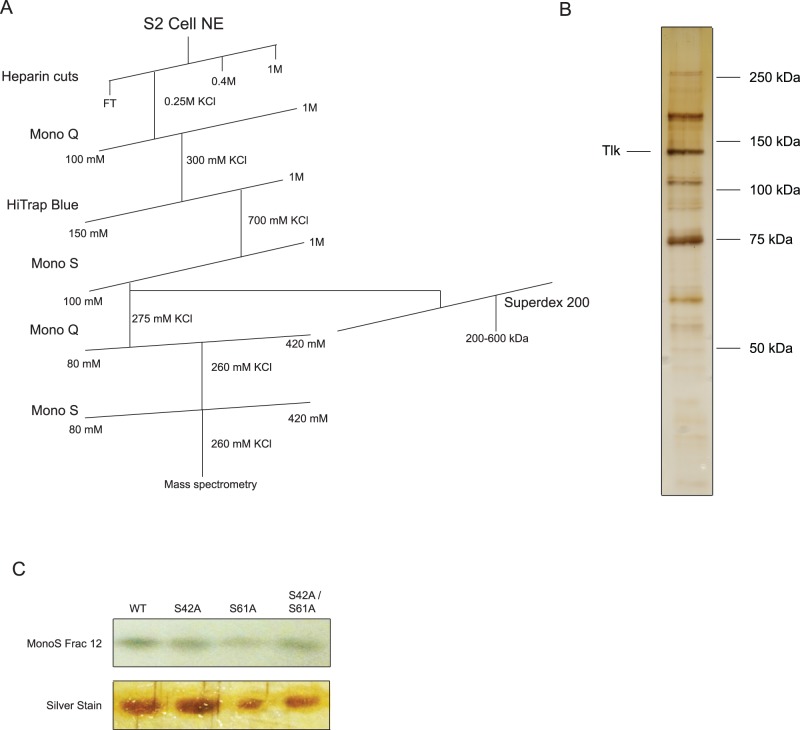
Purification of a second PSI kinase activity. A) Purification strategy for tousled-like (tlk) kinase. B) Protein composition of peak fraction activity from the final Mono S column visualized by silver staining. The species identified as tlk is labeled. C) *In vitro* kinase assay of PSI mutants. Serine to alanine PSI mutants were phosphorylated *in vitro* using the peak fraction of activity from the final Mono S column. Assays were visualized using autoradiography, and, to ensure equal protein loading, silver staining.

Although the majority of the activity in the initial fractionation step resided in the 1 M KCl fraction and was likely due to casein kinase II, we detected a smaller peak of activity in the 250 mM fraction. We further fractionated this peak of activity over several chromatographic columns (Figure 3A). After fractionation, we again visualized the peak activity fraction by silver stain (Figure 3B) and excised prominent bands from the gel and analyzed them by mass spectrometry. We identified tousled-like kinase (tlk) as a component of the prominent band migrating at 130 kDa. No other known protein kinases were identified in this fraction. A second, independent fractionation also identified tlk as the lone kinase in the final peak activity fraction.

We then tested the ability of the tlk-containing fraction to phosphorylate the serine-to-alanine mutant PSI substrates at Ser42 and Ser61 (Figure 3C). Although the tlk-containing fraction did efficiently phosphorylate the wildtype substrate, it also phosphorylated the two mutants. However, there are one threonine and fifteen serine residues in the PSI truncation substrate that may serve as alternate phosphorylation sites for the mutant substrate. No phosphorylation site could be found in the truncated PSI substrate by mass spectrometry, suggesting that the observed activity resulted in very low levels of modification and/or modification distributed over multiple sites.

### PSI Mutants Show Differential Protein Interaction Profiles

PSI contains four RNA-binding KH domains [11], but the identified phosphorylation sites lie N-terminally to them. The phosphorylation sites are, however, near a very glycine-rich region. Glycine-rich regions are known to be mediators of protein-protein interactions, particularly in RNA binding proteins [27]. We therefore hypothesized that the phosphorylation state of PSI may influence its ability to interact with other proteins.

To test this idea, we performed GST pulldowns in *Drosophila* Kc cell nuclear extract using GST-tagged wildtype and mutant N-terminal PSI truncations as bait (Figure 4A). As before, these truncations again consisted of the first 95 amino acids of PSI. We also performed the pulldowns using bait proteins that had been pre-phosphorylated by treatment with casein kinase II and ATP. We then analyzed the PSI-interacting proteins by mass spectrometry.

Interestingly, a 75 kDa protein interacted strongly with the wildtype and S61A PSI N-terminal truncations, but not with the S42A or S42A/S61A PSI truncations (Figure 4A). This protein also seemed to shift in mobility when casein kinase II had been added to the extract. Mass spectrometry analysis revealed several peptides of the 75 kDa Recombination Repair Protein 1 (Rrp1) in the wildtype and S61A pulldowns, but none in the S42A and S42A/S61A pulldowns. Interestingly, Rrp1 also contains two copies of the casein kinase II phosphorylation motif, indicating that its change in mobility when casein kinase II is added to the pulldown may be the result of its own phosphorylation by CKII. The ability of Rrp1 to interact with PSI, then, may depend on the phosphorylation state of Ser 42.

To perform of a more comprehensive analysis of the proteins that interact with PSI, we expressed epitope-tagged full-length PSI in S2 cells. We then immunoprecipitated the exogenous PSI using the polyoma (also known as Glu-Glu) epitope, eluted from this immunoprecipitation using free polyoma (EYMPME) peptide, and treated with RNase A. We then immunoprecipitated the eluate using a polyclonal anti-PSI antibody [28] (Figure 4B, 4C). After eluting PSI from the antibody resin with acidified glycine, we analyzed the PSI-interacting proteins using mass spectrometry (Figure 4D, Supplemental Table 1).

We identified several splicing factors among the interacting proteins, including snRNP70K, which had been previously shown to directly interact with the A/B domain of PSI [8]. These PSI-interacting proteins also included hrp48, a factor known to play a role in the P element splicing silencer [6], [29],the hnRNAP protein hrp59, the splicing factor PUF68, the RNA helicase Rm62 and SR protein Srp54. Rrp1, identified as an interacting partner with truncated PSI, was present but with fewer peptides than proteins listed in Figure 4D. Interestingly, we also identified several cytoskeletal proteins, including actin and the alpha and beta subunits of tubulin. These proteins appear not to be non-specific contaminants, as they did not co-purify with non-specific rabbit IgG antibody done in parallel. Taken together, these proteins appear to associate with PSI via protein-protein interactions in an RNase-insensitive manner and thus might be functioning along with PSI in the processing of specific nuclear pre-mRNAs.

## Discussion

In this study, we have identified two kinases that phosphorylate the splicing factor PSI. Although we could definitively show that the phosphorylation site for casein kinase II was Ser 61, we were unable to definitively show that Ser 42 was phosphorylated by tlk. As very little is known about its preferred motif [30], it is difficult to assess whether tlk can phosphorylate Ser 42. Ser 42 is additionally in close proximity to several other serines, and thus its mutation may only shift phosphorylation to one or more of these nearby sites. However, given that we twice identified tlk as the only kinase present in a fraction that efficiently phosphorylated the PSI substrate, it is likely that tlk can phosphorylate PSI.

These phosphorylation events do not occur in or near the RNA-binding KH domains of PSI and are thus unlikely to modulate RNA-binding activity. They may, however, affect the protein interaction partners of PSI. Using GST pulldowns, we showed that Rrp1 requires the presence of Ser 42 to be able to interact efficiently with PSI. Rrp1 is an exonuclease that is involved in DNA damage repair [31]. Connections between splicing and DNA repair have been previously recognized [32]. Interestingly, human tlk has also been implicated in DNA damage and repair [33], [34], [35]. Furthermore, Ser 42 is conserved between PSI and the human ortholog of PSI, KSRP. Human KSRP is also phosphorylated upon DNA damage [36], further strengthening a possible link between the phosphorylation state of PSI and DNA damage events.

The interaction of PSI with several cytoskeletal proteins was unexpected. PSI is essentially exclusively nuclear [12], and the immunoprecipitations and purifications performed here began with nuclear extracts. Both actin and tubulin, however, are known to exist in the nucleus [37], [38]. Furthermore, certain transcriptional complexes are known to contain actin, and actin has been shown to interact with several hnRNP proteins [39]. The potential importance and role of the interaction of PSI with these cytoskeletal proteins will require further investigation as will a full understanding of the consequences of these two phosphorylation events in PSI.

## Materials and Methods

### Purification of Polyoma-tagged Full-length PSI from Kc Cells

*Drosophila* Kc cells were a gift from the lab of Joan Steitz (Yale University) and were first produced from 12 hour-old embryos by Echalier and Ohanessian in 1969 [40]. Nuclear extract from Kc cells was incubated with 50 µL of anti-polyoma resin for 1 hr at 4°C. The resin was then washed three times with 1 mL IPB2 buffer (20 mM Tris pH 8.0, 2 mM EDTA, 400 mM NaCl, 0.2% NP-40, 1 mM dithiothreitol). The resin was then resuspended in 500 µL of IPB2 and treated with 0.5 µL of RNase A (Promega). The resin was then washed twice with 1 mL IPB2, and protein was eluted off the resin using elution buffer at 65°C (8 M urea/100 mM Tris pH 8.5). The samples were then prepared for mass spectrometry as described below.

### Purification of N-terminal PSI Truncation

A cDNA fragment containing amino acids 2–95 of PSI was cloned into pRSETA between the NdeI and KpnI restriction sites. Additionally, the cDNA contained a His_6_ tag on the N-terminal end and a polyoma (EYMPME) tag on the C-terminal end. The PSI truncation was expressed in BL21(DE3) pLYS E cells and purified using nickel affinity chromatography.

### In vitro Kinase Assays

In vitro kinase assays contained the following: 5 µL fraction to be assayed, 1 µg PSI fragment, 0.5 µL γ-^32^P-ATP (7000 Ci/mmol), 25 mM Hepes-KOH, pH 7.5, 0.5 mM EDTA, 5% glycerol, 5 mM MgCl_2_, 100 mM KCl, 0.5 mM DTT, 0.4 mM PMSF, 10 mM NaF, 5 mM beta-glycerol-phosphate, 25 µM ATP in a final volume of 50 µL. The reactions were incubated at room temperature for 30 min. Ten µL of protein sample buffer were added, and 15 microliters of the sample was then analyzed by SDS-PAGE. The gel was silver-stained, dried, and exposed to X-ray film for two hours to determine the chromatography fractions that contained PSI-phosphorylating activity.

### GST Pulldowns

cDNA fragments for each PSI mutant were cloned into pGEX-2TK and expressed as GST fusions in Rosetta(DE3) pLYS S *E. coli* cells. Lysate containing the overexpressed PSI fusion proteins was incubated with glutathione-sepharose beads for 3 hr at 4°C. The beads were then washed 3 times with Buffer A (50 mM Tris-HCl, pH 8.0, 1 M NaCl, 0.5 mM DTT, 0.4 mM PMSF, 10% glycerol) and 2 times with 1X Casein Kinase II buffer (NEB P6010S). ATP was then added to all samples to 5 mM, and 1 uL purified human casein kinase II (NEB P6010S) was added to those samples that were to be phosphorylated. The samples were then incubated overnight at 30°C.

100 µL Kc nuclear extract was then added, and the beads were incubated at 4°C for 3 hrs. The beads were then washed three times with buffer B (20 mM Tris-HCl, pH 8.0, 200 mM NaCl, 0.02% NP-40, 0.5 mM DTT, 0.4 mM PMSF), and bound proteins were eluted by incubating with 50 µL elution buffer (50 mM Tris-HCl, pH 8.0, 100 mM NaCl, 10% glycerol, 20 mM glutathione, 0.5 mM DTT, 0.4 mM PMSF) at room temperature for 45 min. Bound proteins were analyzed by SDS-PAGE, silver-staining, and mass spectrometry.

### PSI Immunoprecipitation

RNP extract [41], [42] from *Drosophila* S2 cells stably expressing polyoma (also known as Glu-Glu) tagged PSI under the control of the metallothionein promoter was made after inducing expression of the transgene with 200 µM CuSO_4_ for 36 hours. Briefly, cells were swelled in hypotonic buffer and nuclei were isolated using a dounce homogenizer. The nuclei were then sonicated, and the resulting lysate was passed over a 30% sucrose cushion to separate the light nucleoplasm from the dense chromatin-associated fraction.

The nucleoplasm was then collected and incubated at 4°C for 2 hrs with protein-A sepharose beads that had been pre-incubated with anti-polyoma antibody. The beads were then washed 3 times with wash buffer (20 mM Hepes-KOH pH 7.5, 200 mM KCl, 2 mM MgCl_2_, 0.5 mM DTT, 0.05% NP-40, 0.4 mM PMSF). Proteins were then eluted from the resin by two incubations with 200 µL elution buffer (wash buffer supplemented with 100 µg/mL polyoma peptide (EYMPME)).

Elutions were then incubated for 1 hr at 4°C with protein-A Dynabeads (Invitrogen 100-01D) that had been pre-incubated with polyclonal anti-PSI antibody. As a control, the elutions were also incubated with protein-A Dynabeads that had been pre-incubated with rabbit IgG.The beads were then washed three times with wash buffer. Bound proteins were eluted off the beads in two elutions by incubating with 100 µL elution buffer (100 mM glycine, pH 2.5, 100 mM NaCl). The eluates from both the anti-PSI and rabbit IgG beads were analyzed by silver staining, western blotting, and mass spectrometry. Proteins identified as interacting with PSI by having at least 5 detected peptides are listed in Figure 4D.

### Sample Preparation and Mass Spectrometry for Phosphopeptide Analysis

Immunopurified PSI was adjusted to 40% methanol, 100 mM ammonium bicarbonate pH8.5, 5 mM TCEP, 1.5% ProteaseMAX (Promega) and subjected to carboxyamidomethylation of cysteines. Two samples were created and analyzed separately and then the results were combined. The first sample was digested with trypsin and chymotrypsin. The second sample was divided, and one portion was digested with a combination of trypsin and chymotrypsin. The other portion was digested with thermolysin. The two fractions were then recombined for analysis. All digestions were incubated overnight at 37°C and stopped by the addition of 5% formic acid. A 3 phase nano LC column was packed in a 100 µm inner diameter glass capillary with an emitter tip. The column consisted of 10 cm of Polaris C18 5 µm packing material (Varian), followed by 4 cm of Partisphere 5 SCX (Whatman), followed by another 2 cm of Polaris C18. The column was loaded by use of a pressure bomb and washed extensively with buffer A (see below). The column was then directly coupled to an electrospray ionization source mounted on a Thermo-Fisher LTQ XL linear ion trap mass spectrometer. Data collection was programmed so that neutral loss of phosphate would tigger the collection of an MS3 spectrum of the neutral loss peak. An Agilent 1200 HPLC equipped with a split line so as to deliver a flow rate of 30 nl/min was used for chromatography. Peptides were eluted using a 4-step MudPIT procedure [43]. Buffer A was 5% acetonitrile/0.02% heptaflurobutyric acid (HBFA); buffer B was 80% acetonitrile/0.02% HBFA. Buffer C was 250 mM ammonium acetate/5% acetonitrile/0.02% HBFA; buffer D was same as buffer C, but with 500 mM ammonium acetate. The programs SEQUEST and DTASELECT were used to identify peptides and proteins from the Drosophila database [44], [45]. Phosphopeptides were confirmed by manual inspection of the spectra.

### Sample Preparation and Mass Spectrometry for Protein Interaction Analysis

The protein solution was adjusted to 8 M urea, subjected to carboxyamidomethylation of cysteines, and digested with trypsin. The sample was then desalted using a C18 spec tip (Varian). A 2 phase nano LC column was packed and loaded as described above. The column consisted of 10 cm of Polaris C18 5 µm packing material (Varian), followed by 4 cm of Partisphere 5 SCX (Whatman). Chromatography, mass spectrometry and data analysis were as described above, except that no MS3 spectra were collected.

### Mass Spectrometry of Gel Bands

Excised gel bands were treated to cause carboxyamidomethylation of cysteines, digested with trypsin and the resulting peptides extracted. Samples were loaded on a 100 micromolar ID, 10 cm column of Polaris C18 and analyzed by LC-MS/MS with a linear gradient consisting of buffer A and buffer B as above.

## Supporting Information

Figure S1 **Identification of phosphorylation sites by mass spectrometry.** A and B) MS3 spectra of phosphopeptides. B and Y series ions and loss of water are indicated. Inset: sequence of the phosphopeptide and SEQUEST statistics. C and D) MS2 spectra of corresponding unmodified peptides.
(EPS)

Table S1 **Protein-protein interactions of PSI.** Proteins identified with at least two peptides as copurifying in pulldowns of full length PSI. SEQUEST Xcorr is listed for each peptide.
(XLS)

## References

[1] Wang ET, Sandberg R, Luo S, Khrebtukova I, Zhang L, et al (2008) Alternative isoform regulation in human tissue transcriptomes. Nature 456: 470–476. 18978772 10.1038/nature07509 PMC2593745

[2] Graveley BR, Brooks AN, Carlson JW, Duff MO, Landolin JM, et al (2011) The developmental transcriptome of Drosophila melanogaster. Nature 471: 473–479. 21179090 10.1038/nature09715 PMC3075879

[3] Ben-Dov C, Hartmann B, Lundgren J, Valcarcel J (2008) Genome-wide analysis of alternative pre-mRNA splicing. J Biol Chem 283: 1229–1233. 18024428 10.1074/jbc.R700033200

[4] Pan Q, Shai O, Lee LJ, Frey BJ, Blencowe BJ (2008) Deep surveying of alternative splicing complexity in the human transcriptome by high-throughput sequencing. Nat Genet 40: 1413–1415. 18978789 10.1038/ng.259

[5] Blanchette M, Green RE, Brenner SE, Rio DC (2005) Global analysis of positive and negative pre-mRNA splicing regulators in Drosophila. Genes Dev 19: 1306–1314. 15937219 10.1101/gad.1314205 PMC1142554

[6] Siebel CW, Kanaar R, Rio DC (1994) Regulation of tissue-specific P-element pre-mRNA splicing requires the RNA-binding protein PSI. Genes Dev 8: 1713–1725. 7958851 10.1101/gad.8.14.1713

[7] Adams MD, Tarng RS, Rio DC (1997) The alternative splicing factor PSI regulates P-element third intron splicing in vivo. Genes Dev 11: 129–138. 9000056 10.1101/gad.11.1.129

[8] Labourier E, Adams MD, Rio DC (2001) Modulation of P-element pre-mRNA splicing by a direct interaction between PSI and U1 snRNP 70 K protein. Mol Cell 8: 363–373. 11545738 10.1016/s1097-2765(01)00311-2

[9] Ignjatovic T, Yang JC, Butler J, Neuhaus D, Nagai K (2005) Structural basis of the interaction between P-element somatic inhibitor and U1–70 k essential for the alternative splicing of P-element transposase. J Mol Biol 351: 52–65. 15990112 10.1016/j.jmb.2005.04.077

[10] Siebel CW, Fresco LD, Rio DC (1992) The mechanism of somatic inhibition of Drosophila P-element pre-mRNA splicing: multiprotein complexes at an exon pseudo-5′ splice site control U1 snRNP binding. Genes Dev 6: 1386–1401. 1322855 10.1101/gad.6.8.1386

[11] Amarasinghe AK, MacDiarmid R, Adams MD, Rio DC (2001) An in vitro-selected RNA-binding site for the KH domain protein PSI acts as a splicing inhibitor element. RNA 7: 1239–1253. 11565747 10.1017/s1355838201010603 PMC1370169

[12] Labourier E, Blanchette M, Feiger JW, Adams MD, Rio DC (2002) The KH-type RNA-binding protein PSI is required for Drosophila viability, male fertility, and cellular mRNA processing. Genes Dev 16: 72–84. 11782446 10.1101/gad.948602 PMC155316

[13] Anko ML, Muller-McNicoll M, Brandl H, Curk T, Gorup C, et al (2012) The RNA-binding landscapes of two SR proteins reveal unique functions and binding to diverse RNA classes. Genome Biol 13: R17. 22436691 10.1186/gb-2012-13-3-r17 PMC3439968

[14] Lareau LF, Inada M, Green RE, Wengrod JC, Brenner SE (2007) Unproductive splicing of SR genes associated with highly conserved and ultraconserved DNA elements. Nature 446: 926–929. 17361132 10.1038/nature05676

[15] Xiao SH, Manley JL (1998) Phosphorylation-dephosphorylation differentially affects activities of splicing factor ASF/SF2. EMBO J 17: 6359–6367. 9799243 10.1093/emboj/17.21.6359 PMC1170960

[16] Wang X, Bruderer S, Rafi Z, Xue J, Milburn PJ, et al (1999) Phosphorylation of splicing factor SF1 on Ser20 by cGMP-dependent protein kinase regulates spliceosome assembly. EMBO J 18: 4549–4559. 10449420 10.1093/emboj/18.16.4549 PMC1171529

[17] Boudrez A, Beullens M, Waelkens E, Stalmans W, Bollen M (2002) Phosphorylation-dependent interaction between the splicing factors SAP155 and NIPP1. J Biol Chem 277: 31834–31841. 12105215 10.1074/jbc.M204427200

[18] Roth MB, Murphy C, Gall JG (1990) A monoclonal antibody that recognizes a phosphorylated epitope stains lampbrush chromosome loops and small granules in the amphibian germinal vesicle. J Cell Biol 111: 2217–2223. 1703534 10.1083/jcb.111.6.2217 PMC2116404

[19] Xiao SH, Manley JL (1997) Phosphorylation of the ASF/SF2 RS domain affects both protein-protein and protein-RNA interactions and is necessary for splicing. Genes Dev 11: 334–344. 9030686 10.1101/gad.11.3.334

[20] Zhai B, Villen J, Beausoleil SA, Mintseris J, Gygi SP (2008) Phosphoproteome analysis of Drosophila melanogaster embryos. J Proteome Res 7: 1675–1682. 18327897 10.1021/pr700696a PMC3063950

[21] Glover CV, Shelton ER, Brutlag DL (1983) Purification and characterization of a type II casein kinase from Drosophila melanogaster. J Biol Chem 258: 3258–3265. 6298230

[22] Bourbon HM, Martin-Blanco E, Rosen D, Kornberg TB (1995) Phosphorylation of the Drosophila engrailed protein at a site outside its homeodomain enhances DNA binding. J Biol Chem 270: 11130–11139. 7744743 10.1074/jbc.270.19.11130

[23] Jaffe L, Ryoo HD, Mann RS (1997) A role for phosphorylation by casein kinase II in modulating Antennapedia activity in Drosophila. Genes Dev 11: 1327–1340. 9171376 10.1101/gad.11.10.1327

[24] Packman LC, Kubota K, Parker J, Gay NJ (1997) Casein kinase II phosphorylates Ser468 in the PEST domain of the Drosophila IkappaB homologue cactus. FEBS Lett 400: 45–50. 9000511 10.1016/s0014-5793(96)01324-5

[25] Willert K, Brink M, Wodarz A, Varmus H, Nusse R (1997) Casein kinase 2 associates with and phosphorylates dishevelled. EMBO J 16: 3089–3096. 9214626 10.1093/emboj/16.11.3089 PMC1169927

[26] Kemp BE, Pearson RB (1990) Protein kinase recognition sequence motifs. Trends Biochem Sci 15: 342–346. 2238044 10.1016/0968-0004(90)90073-k

[27] Wang J, Dong Z, Bell LR (1997) Sex-lethal interactions with protein and RNA. Roles of glycine-rich and RNA binding domains. J Biol Chem 272: 22227–22235. 9268369 10.1074/jbc.272.35.22227

[28] Siebel CW, Admon A, Rio DC (1995) Soma-specific expression and cloning of PSI, a negative regulator of P element pre-mRNA splicing. Genes Dev 9: 269–283. 7867926 10.1101/gad.9.3.269

[29] Hammond LE, Rudner DZ, Kanaar R, Rio DC (1997) Mutations in the hrp48 gene, which encodes a Drosophila heterogeneous nuclear ribonucleoprotein particle protein, cause lethality and developmental defects and affect P-element third-intron splicing in vivo. Mol Cell Biol 17: 7260–7267. 9372958 10.1128/mcb.17.12.7260 PMC232583

[30] Pilyugin M, Demmers J, Verrijzer CP, Karch F, Moshkin YM (2009) Phosphorylation-mediated control of histone chaperone ASF1 levels by Tousled-like kinases. PLoS One 4: e8328. 20016786 10.1371/journal.pone.0008328 PMC2791443

[31] Szakmary A, Huang SM, Chang DT, Beachy PA, Sander M (1996) Overexpression of a Rrp1 transgene reduces the somatic mutation and recombination frequency induced by oxidative DNA damage in Drosophila melanogaster. Proc Natl Acad Sci U S A 93: 1607–1612. 8643678 10.1073/pnas.93.4.1607 PMC39989

[32] Chaouki AS, Salz HK (2006) Drosophila SPF45: a bifunctional protein with roles in both splicing and DNA repair. PLoS Genet 2: e178. 17154718 10.1371/journal.pgen.0020178 PMC1687153

[33] Carrera P, Moshkin YM, Gronke S, Sillje HH, Nigg EA, et al (2003) Tousled-like kinase functions with the chromatin assembly pathway regulating nuclear divisions. Genes Dev 17: 2578–2590. 14561777 10.1101/gad.276703 PMC218151

[34] Krause DR, Jonnalagadda JC, Gatei MH, Sillje HH, Zhou BB, et al (2003) Suppression of Tousled-like kinase activity after DNA damage or replication block requires ATM, NBS1 and Chk1. Oncogene 22: 5927–5937. 12955071 10.1038/sj.onc.1206691

[35] Groth A, Lukas J, Nigg EA, Sillje HH, Wernstedt C, et al (2003) Human Tousled like kinases are targeted by an ATM- and Chk1-dependent DNA damage checkpoint. EMBO J 22: 1676–1687. 12660173 10.1093/emboj/cdg151 PMC152895

[36] Matsuoka S, Ballif BA, Smogorzewska A, McDonald ER 3rd, Hurov KE, et al (2007) ATM and ATR substrate analysis reveals extensive protein networks responsive to DNA damage. Science 316: 1160–1166. 17525332 10.1126/science.1140321

[37] Menko AS, Tan KB (1980) Nuclear tubulin of tissue culture cells. Biochim Biophys Acta 629: 359–370. 7388040 10.1016/0304-4165(80)90108-7

[38] Olave IA, Reck-Peterson SL, Crabtree GR (2002) Nuclear actin and actin-related proteins in chromatin remodeling. Annu Rev Biochem 71: 755–781. 12045110 10.1146/annurev.biochem.71.110601.135507

[39] Zheng B, Han M, Bernier M, Wen JK (2009) Nuclear actin and actin-binding proteins in the regulation of transcription and gene expression. FEBS J 276: 2669–2685. 19459931 10.1111/j.1742-4658.2009.06986.x PMC2978034

[40] Echalier G, Ohanessian A (1969) Isolation, in tissue culture, of Drosophila melanogaster cell lines. C R Acad Sci Hebd Seances Acad Sci D 268: 1771–1773. 4976834

[41] Pinol-Roma S, Choi YD, Dreyfuss G (1990) Immunological methods for purification and characterization of heterogeneous nuclear ribonucleoprotein particles. Methods Enzymol 181: 317–325. 2143256 10.1016/0076-6879(90)81132-e

[42] Pinol-Roma S, Swanson MS, Matunis MJ, Dreyfuss G (1990) Purification and characterization of proteins of heterogeneous nuclear ribonucleoprotein complexes by affinity chromatography. Methods Enzymol 181: 326–331. 2143257 10.1016/0076-6879(90)81133-f

[43] Washburn MP, Wolters D, Yates JR 3rd (2001) Large-scale analysis of the yeast proteome by multidimensional protein identification technology. Nat Biotechnol 19: 242–247. 11231557 10.1038/85686

[44] Eng JM, McCormack AL, Yates JR (1994) An approach to correlate tandem mass spectral data of peptides with amino acid sequences in a protein database. Journal of the American Society for Mass Spectrometry 5: 976–989. 24226387 10.1016/1044-0305(94)80016-2

[45] Tabb DL, McDonald WH, Yates JR 3rd (2002) DTASelect and Contrast: tools for assembling and comparing protein identifications from shotgun proteomics. J Proteome Res 1: 21–26. 12643522 10.1021/pr015504q PMC2811961

